# Supplementary material for: Adaptation of Bacillus subtilis MreB Filaments to Osmotic Stress Depends on Influx of Potassium Ions
Source: Microorganisms. 2024 Jun 27;12(7):1309. doi: 10.3390/microorganisms12071309 (PMC11279060; doi:10.3390/microorganisms12071309)
Supplement: Supplementary file 1 [file microorganisms-12-01309-s001.zip › microorganisms-3018098-supplementary.pdf]

Supplementary Data Dersch and Graumann 2024

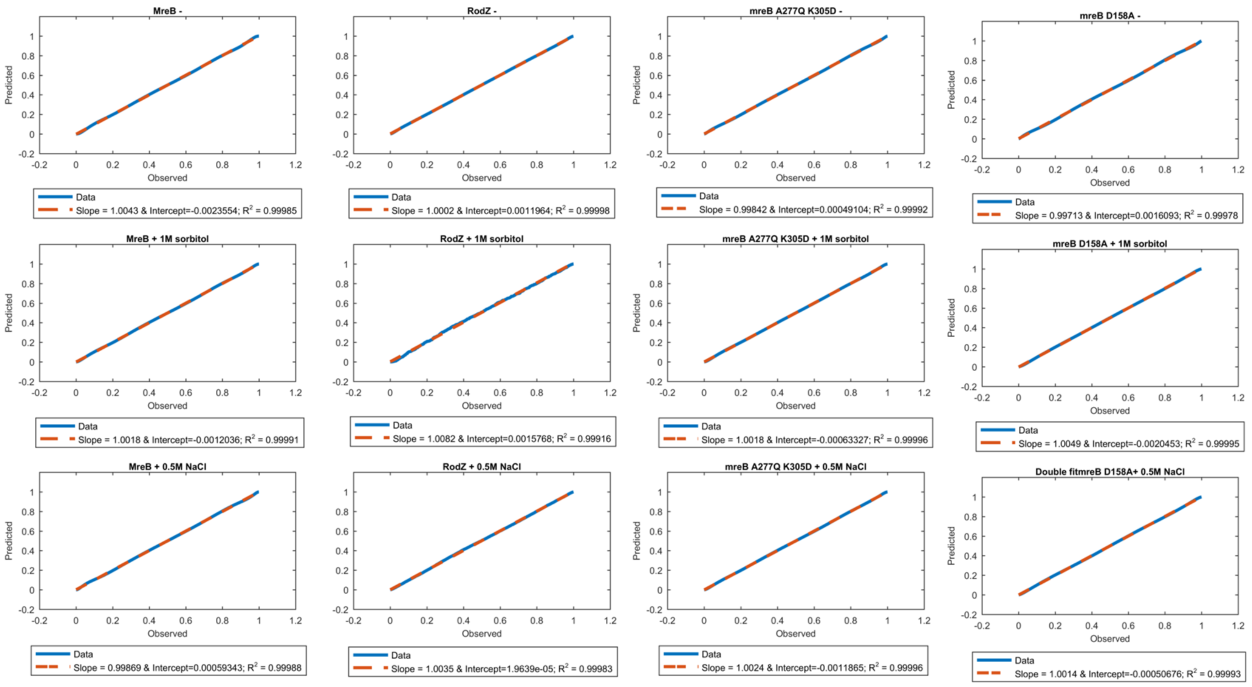

Fig. S1: Goodness of fit for two population Gaussian-mixture-model fit of MreB, RodZ, MreB A277Q/K305D and MreB D158A displacements under various growth conditions.

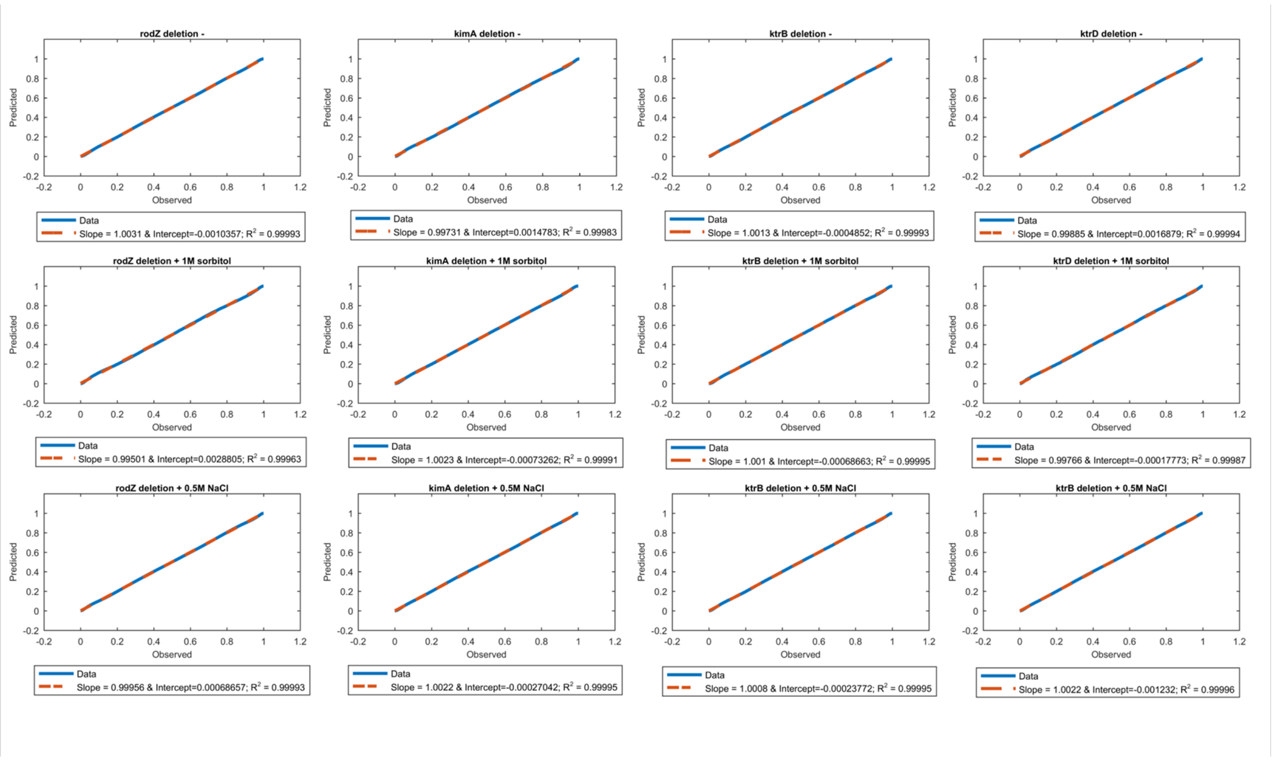

Fig. S2: Goodness of fit for two population Gaussian-mixture-model fit of MreB displacements, tracked in a *ΔrodZ, ΔkimA, ΔktrB* and *ΔktrD* background, under various growth conditions.

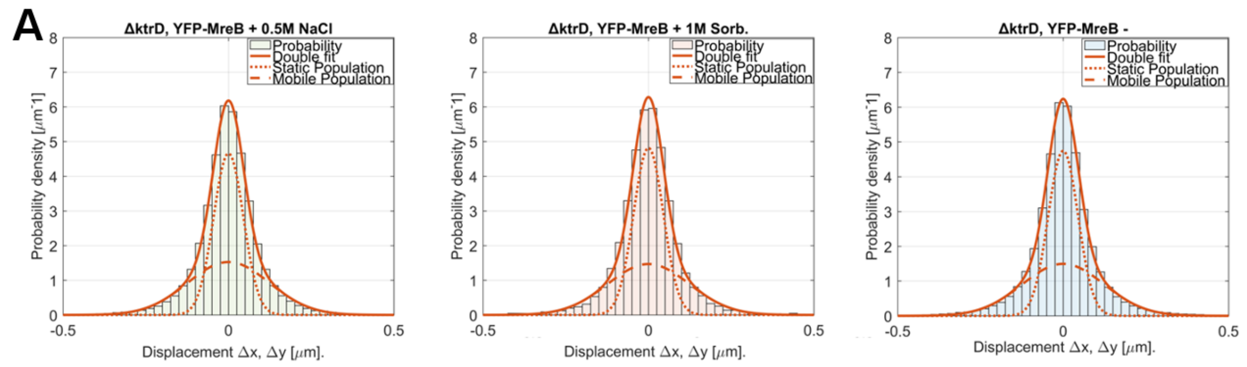

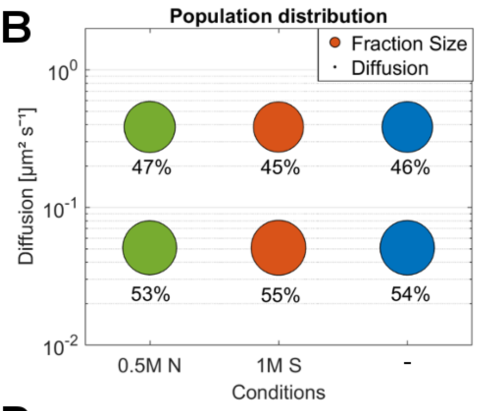

C

	0.5M NaCl	1M Sorbitol	-
Static D ± sd [µm² s⁻¹]	0.051 ± 0.00075	0.051 ± 0.00075	0.051 ± 0.00075
Mobile D ± sd [µm² s⁻¹]	0.38 ± 0.0077	0.38 ± 0.0077	0.38 ± 0.0077
Static Fraction ± sd [%]	53 ± 0.068	55 ± 0.027	54 ± 0.065
Mobile Fraction ± sd [%]	47 ± 0.068	45 ± 0.027	46 ± 0.065

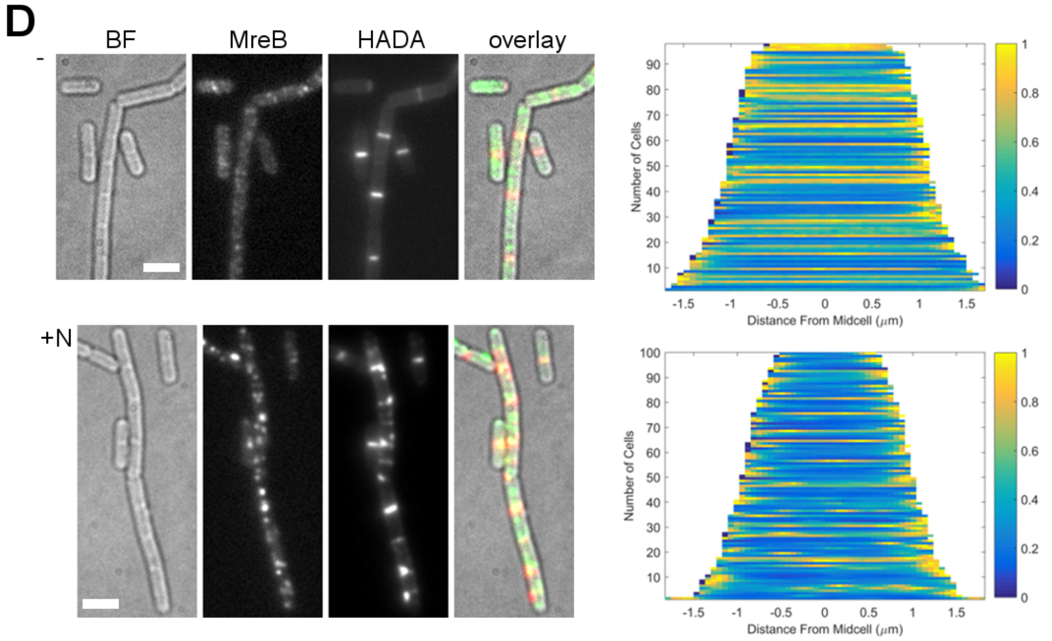

Fig. S3: Adaption of MreB dynamics in response to osmotic stress in a *ΔktrD* background. **A:** Two-population Gaussian-mixture-model (GMM) fit of displacement vs. probability density for YFP-MreB under the control of the xylose promotor (+0.01% xylose) in a *ΔktrB* background, in $S7_{50}$ minimal media under normal growth conditions (-) and with the addition of 500 mM NaCl (+N) or 1 M sorbitol (+S); **B:** Bubble-plot of the diffusive populations (relative fraction sizes, D [µm^2 s– 1]) as identified by GMM curve fit in A; **C:** Corresponding table of diffusion and relative fraction sizes for the slow-mobile and mobile populations; **D:**

Bright field (BF), YFP-MreB (green-channel), HADA (red-channel, 0.5 mM) and overlay images of *B. subtilis* cells (*ΔktrB*) in exponential phase, expressing YFP-MreB under the control of the xylose promotor (+0.01% xylose) in S7$_{50}$ media and corresponding demograph of the distribution of HADA signal throughout n = 100 cells (20 min HADA staining without stress (-) and with added 0.5 M NaCl, images taken after washing 3 times with PBS), scalebar 2 µm.

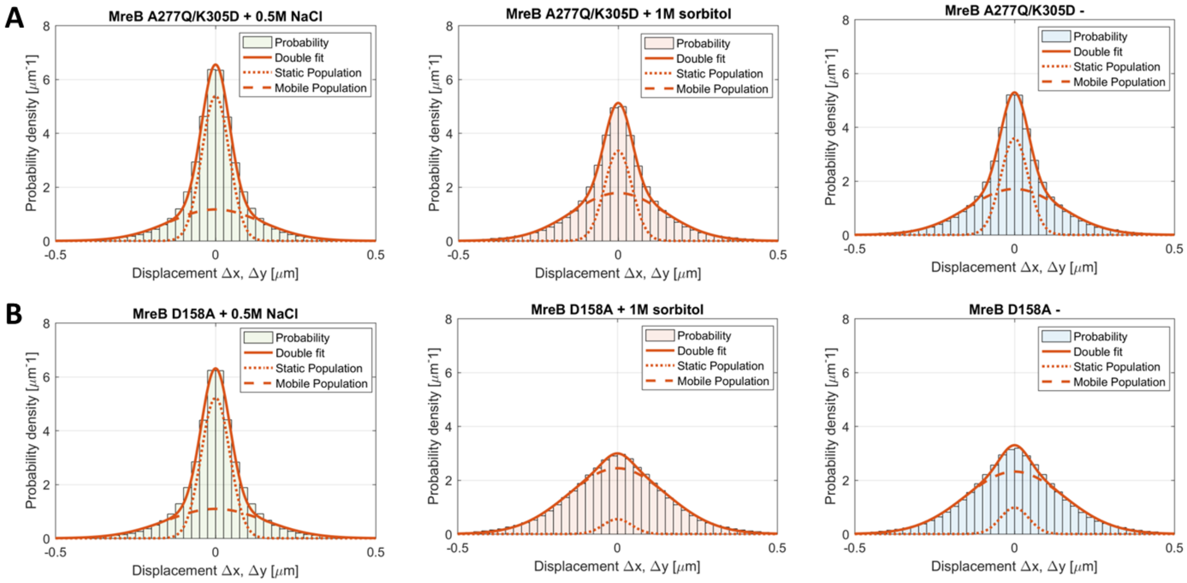

C

	0.5M NaCl	1M Sorbitol	WT
Static D ± sd [µm² s⁻¹]	0.046 ± 0.000036	0.046 ± 0.000036	0.046 ± 0.000036
Mobile D ± sd [µm² s⁻¹]	0.52 ± 0.00036	0.52 ± 0.00036	0.52 ± 0.00036
Static Fraction ± sd [%]	58 ± 0.027	36 ± 0.027	39 ± 0.027
Mobile Fraction ± sd [%]	42 ± 0.027	64 ± 0.027	61 ± 0.027

D

	0.5M NaCl	1M Sorbitol	WT
Static D ± sd [µm² s⁻¹]	0.049 ± 0.000053	0.049 ± 0.000053	0.049 ± 0.000053
Mobile D ± sd [µm² s⁻¹]	0.59 ± 0.00028	0.59 ± 0.00028	0.59 ± 0.00028
Static Fraction ± sd [%]	58 ± 0.029	6.1 ± 0.024	11 ± 0.023
Mobile Fraction ± sd [%]	42 ± 0.029	94 ± 0.024	89 ± 0.023

Fig. S4: Two-population Gaussian-mixture-model (GMM) fit of displacement vs. probability density for MreB A277Q/K305D (**A**) and MreB D158A (**B**) and calculated diffusion values and fraction sizes (**C+D**) for unstressed cells (-), and cell growing with added 0.5M Nacl or 1M sorbitol.
